# Periodontitis and Systemic Disease: The Impact of Covariate Selection

**DOI:** 10.1177/00220345251356469

**Published:** 2025-07-17

**Authors:** N.Z. Bashir, B.A.R. Woolf, S. Burgess, E. Bernabé

**Affiliations:** 1 MRC Biostatistics Unit, University of Cambridge, Cambridge, UK; 2 MRC Integrative Epidemiology Unit, University of Bristol, Bristol, UK; 3 School of Psychological Science, University of Bristol, Bristol, UK; 4 Cardiovascular Epidemiology Unit, University of Cambridge, Cambridge, UK; 5 Institute of Dentistry, Queen Mary University of London, London, UK

**Keywords:** Cardiovascular diseases, Cognitive function, Epidemiology, Periodontal diseases, Statistics, Statistical model

## Abstract

Most evidence implicating periodontitis as a causal agent in systemic disease comes from observational research, where confounding is ubiquitous. There are substantial methodological differences among observational studies, of which an important one is the choice of covariates used to adjust for confounding. The present study assesses the impact of covariate selection on the association between periodontitis and systemic disease, using cardiovascular disease and cognitive function as examples. Data were taken from the National Health and Nutrition Examination Survey, where periodontal status was assessed by full-mouth examination. Nineteen covariates were available for selection, including sociodemographic factors, health behaviors, and physiologic measures. Multiverse analysis and specification curves were used to visualize how the distribution of estimated associations between periodontitis and systemic disease vary across 393,216 models, depending on the selection of covariates. The findings show that estimates of the association between periodontitis and systemic disease are sensitive to the choice of covariates included within models, in some cases spanning positive and negative directions (i.e., Janus effect). Depending on the covariates included in the adjustment set, the odds ratio for the association of severe periodontitis with cardiovascular disease ranged from 0.81 to 1.51, while the linear regression coefficient for the association with cognitive function ranged from −1.56 to 0.50. Our study makes 2 important contributions. First, we introduce the idea of multiverse analysis to periodontal research and show that it can be a valuable tool for understanding challenges around model selection and misspecification. Second, we use rigorous quantitative methods to highlight the importance of careful covariate selection in determining our understanding of the relationship between periodontitis and systemic disease.

## Introduction

A substantial portion of oral health research is focused on the relationship between periodontal and systemic health, with most evidence coming from observational studies. Based on this, it has been claimed that periodontitis is an important determinant in the risk of developing systemic diseases (Preshaw et al. 2012; Sanz et al. 2020; Chapple et al. 2024).

A key challenge in this field is substantial methodological differences among studies. There has been considerable work on measurement error due to the use of partial- versus full-mouth examination protocols (Akinkugbe et al. 2015; Alshihayb et al. 2020) and differences in case definitions of periodontitis (Manau et al. 2008; Alhassani 2023). The present work focuses on the selection of covariates, which are used to adjust estimates for confounding. The concern is that groups of people with and without periodontitis may systematically differ from one another in a way that is also connected to the systemic disease of interest. Many primary studies do not justify the choice of covariates used to control for confounding, resulting in studies of similar systemic health outcomes adjusting for markedly different covariate sets (Chen et al. 2017; Choi et al. 2019; Nascimento et al. 2023). This is an issue that then permeates systematic reviews, as many published reviews do not state the basic set of covariates that primary studies included or their suitability for pooling in a meta-analysis. Concerns about covariate selection, residual confounding, and model variability have been discussed (Beck and Offenbacher 2002; Baelum and Lopez 2003; Demmer and Desvarieux 2006). In effect, bias related to the selection of covariates may play a substantial role in the interpretation of studies on the periodontal-systemic disease relationship, but the extent of this problem is currently unclear. What is missing in the literature is a systematic, quantitative, and reproducible framework to visualize instability in estimates, thereby strengthening the empirical foundation for long-standing theoretical concerns.

Investigators working in meta-research have formally probed the issue of covariate selection. Depending on the field, the terminology differs, including multiverse analysis, specification curve analysis, garden of forking paths, and the vibration of effects (Steegen et al. 2016; Lonsdorf et al. 2019; Simonsohn et al. 2020; Vinatier et al. 2025). The premise is simple: anytime that investigators choose to adjust or not adjust for a covariate in their model, the number of models that they could have fit grows exponentially. Of the possible models, usually only 1 will be fit, and these are the results that are reported. If the estimates across all possible models would not have differed greatly from one another, we say that the results are stable. Stability of results instills some confidence that model misspecification is not inducing substantial bias.

The multiverse analysis approach is to fit all possible models and then compare the results. If the estimates are widely dispersed, one should be more cautious in the interpretation of significant findings, as these can be more easily attributed to the investigator having simply picked a combination of covariates that yielded a statistically significant result. Such *P* hacking undermines epidemiologic research. Also concerning is when the instability is so substantial that it is possible to obtain estimates that point in opposite directions, depending on how the model is specified. The term *Janus effect* has been used in the literature to describe the scenario when the estimated associations can be positive or negative, depending on which variables are adjusted for (Vinatier et al. 2025). This is concerning because an investigator could reach the conclusion that periodontitis either increases or decreases the risk of developing a systemic disease, depending on which covariates were included in the model.

The aim of this article is to investigate the stability of periodontal-systemic disease associations with respect to covariate selection, by application of a multiverse analysis framework.

## Materials and Methods

### Data Source

We extracted and combined National Health and Nutrition Examination Survey demographic, examination, laboratory, and questionnaire data for the 2011–2012 and 2013–2014 cycles. These multistage cross-sectional surveys are designed to take a representative sample of the noninstitutionalized US population (Chen et al. 2020). Participants are interviewed at home and then invited to a mobile examination center for further interviews, tests, and examinations. These examinations include an oral examination conducted by state-licensed dental practitioners.

### Measures

We assessed 2 systemic health outcomes: cardiovascular disease (CVD) and cognitive function. Individuals were classified as having CVD if they reported a previous medical diagnosis of coronary heart disease, heart attack or myocardial infarction, or stroke. Cognitive function was measured in individuals aged ≥60 y by the Consortium to Establish a Registry for Alzheimer’s Disease (CERAD) test (Fillenbaum et al. 2008). The CERAD test comprises 3 learning tests and 1 delayed recall test, each of which has a maximum score of 10 points. The final CERAD score is the sum of the 4 test scores, ranging from 0 to 40. Higher CERAD scores indicate better cognitive function. All analyses were restricted to those individuals who had either reported a CVD status or took the CERAD test.

We classified periodontal status for participants using case definitions per the Centers for Disease Control and Prevention and the American Academy of Periodontology for population-based surveillance of periodontitis. Dentate individuals were categorized as having no periodontitis (reference) or mild, moderate, or severe periodontitis (Eke et al. 2012). No periodontitis is defined as the absence of mild, moderate, or severe periodontitis. Mild periodontitis is defined as ≥2 interproximal sites with clinical attachment loss (CAL) ≥3 mm and ≥2 interproximal sites with probing pocket depth (PPD) ≥4 mm (not on same tooth) or 1 site with PPD ≥5 mm. Moderate periodontitis is defined as ≥2 interproximal sites with CAL ≥4 mm (not on same tooth) or ≥2 interproximal sites with PPD ≥5 mm (not on same tooth). Severe periodontitis is defined as ≥2 interproximal sites with CAL ≥6 mm (not on same tooth) and ≥1 interproximal site with PPD ≥5 mm. Edentulous individuals were excluded.

We considered 19 covariates as our set of possible adjustments. There is no consensus on which variables should be adjusted for when assessing periodontitis as an exposure, so we selected a broad range of plausible variables for this evaluation. Since age and sex are well-known risk factors that are universally adjusted for in epidemiologic analyses, we kept these in all models as baseline variables, treating age as a continuous variable (in years and top coded at 80) and sex as a categorical variable (men, women). This left the remaining 17 variables to be used as our set of varying adjustments. Categorical variables were as follows: race and ethnicity (non-Hispanic White, non-Hispanic Black, Mexican American, other), educational attainment (below, at, and above high school), marital status (married or living as married, separated, and never married), smoking status (never, former, and current smoker), health insurance (insured, uninsured), and self-reported history of diabetes (whether one had received a diagnosis of diabetes from a medical professional). Continuous variables were as follows: poverty-income ratio (defined as the ratio between household income and the federal poverty threshold; i.e., a value <1.0 indicates that a family is living in poverty), alcohol consumption (drinks per day; a drink was defined as a 12-oz beer, a 4-oz glass of wine, or 1.5 oz of liquor), physical activity (metabolic equivalent hours per week), body mass index, systolic and diastolic blood pressure, glycated hemoglobin (percentage HbA_1c_), total and high-density lipoprotein cholesterol, vitamin D (25-OH D_2_ + 25-OH D_3_), and systemic immune-inflammation index. Systolic and diastolic blood pressure were averaged across 3 separate measures. The systemic immune-inflammation index is computed as *P* × *N*/*L*, where *P*, *N*, and *L* are the peripheral platelet, neutrophil, and lymphocyte counts, respectively; it has been used as a marker of systemic inflammation in the study of chronic disease (Hu et al. 2014; Ma and Li 2023). When cognitive function was assessed as an outcome, history of CVD (as previously defined) was also included in the list of covariates, bringing the total to 18.

### Statistical Analysis

The total number of possible covariate adjustment sets is 131,072 (2^17^) for CVD and 262,144 (2^18^) for cognitive function. We extracted the estimated regression coefficient for the periodontitis exposure, with its associated 95% CI and *P* value. All analyses were complete-case analyses with respect to covariate combination, which adjusted for the complex sample survey design through use of the probability sampling weights, pseudo-primary sampling units, and pseudo-strata. For CVD, the estimated association was the log(odds ratio [OR]) from logistic regression. For cognitive function, the estimated association was the regression coefficient from linear regression.

We visualized the results from multiverse analysis by creating volcano plots of estimated associations against the –log_10_(*P* value). We declared the presence of a Janus effect if the 1st and 99th percentiles of the estimated associations took opposite signs (Vinatier et al. 2025). To evaluate how specific covariates affected the analyses, we plotted specification curves (Simonsohn et al. 2020). Specification curves show the distribution of estimated associations, as well as a binary inclusion matrix highlighting the combination of covariates that were adjusted for to produce a given estimate. They present the estimated associations for every possible combination of covariates. Beneath each estimate is a matrix that indicates whether a given covariate was included in the model to obtain that estimate. This allows for assessment of which covariates may be most influential in determining the stability of the models.

We carried out sensitivity analyses where we replicated the main analyses but instead assessed periodontitis as a continuous exposure in the form of mean CAL across all inspected sites (mean CAL).

## Results

A full description of the included participants is presented in Appendix Tables 1 and 2. A flowchart showing the procedure for selecting participants is presented in Appendix Figure 1.

### Multiverse Analysis

#### Cardiovascular Disease

**Figure 1.**
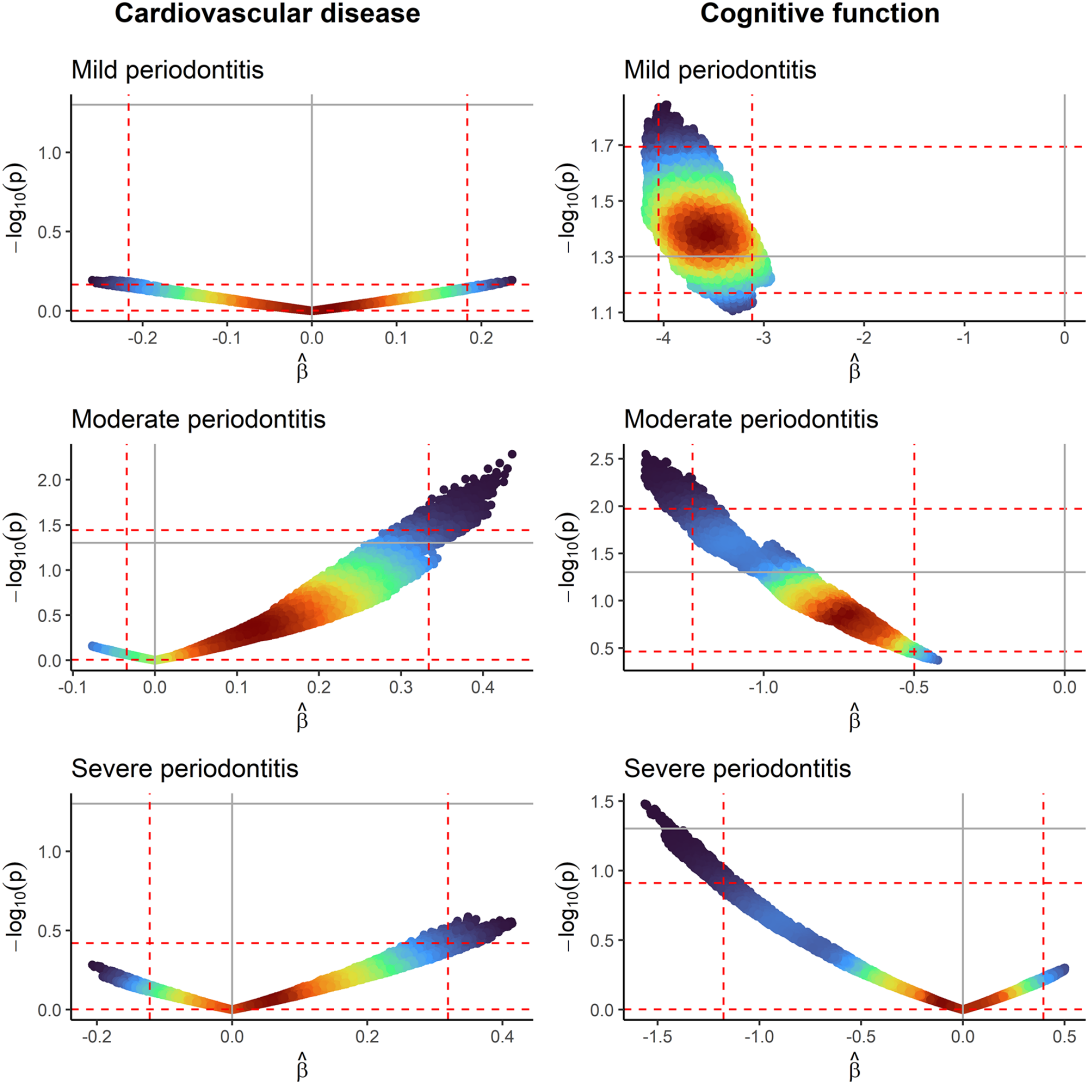
Volcano plots where each point represents the estimated regression coefficient and –log_10_(*P* value) from 1 model, colored by its density (red = high, blue = low). Vertical and horizontal dashed red lines represent the 1st and 99th percentiles of the estimated regression coefficients and –log_10_(*P* value), respectively. Vertical and horizontal solid gray lines represent the null effect and nominal threshold of significance (*P* = 0.05). No periodontitis is the reference category. Left column: x-axes in cardiovascular disease volcano plots are measuring the log(odds ratio). Right column: x-axes in cognitive function volcano plots are measuring the linear regression coefficient. All models are adjusted for age and sex.

The results of the multiverse analyses are presented in Figure 1 (edentulism and mean CAL results are presented in Appendix Fig. 2).

For CVD, a Janus effect is observed in the distribution of estimated associations for all severities of periodontitis. The proportion of negative estimated associations (OR <1) were 50.3% for mild periodontitis, 5.6% for moderate, and 29.3% for severe. The estimates ranged from 0.77 to 1.27 for mild periodontitis, 0.93 to 1.55 for moderate, and 0.81 to 1.51 for severe. In all, none of the estimates for mild periodontitis, 2.1% for moderate, and none for severe had a corresponding *P* < 0.05.

As an example of the Janus effect, adjusting for the following variables results in a positive estimated association of moderate periodontitis with CVD (OR, 1.35, 95% CI, 1.02 to 1.78): age, sex, race and ethnicity, educational attainment, physical activity, health insurance status, HbA_1c_, total and high-density lipoprotein cholesterol, and vitamin D. Yet, adjusting for age, sex, race and ethnicity, educational attainment, poverty-income ratio, smoking status, alcohol consumption, body mass index, diastolic blood pressure, and HbA_1c_ results in a negative estimated association (OR, 0.96; 95% CI, 0.66 to 1.40).

#### Cognitive Function

For cognitive function, the distribution of estimated associations differs depending on the severity of periodontitis. Mild and moderate periodontitis showed a consistently negative association, while a Janus effect was observed with severe periodontitis. The proportion of negative estimated associations (β < 0) were 100% for mild and moderate periodontitis and 60.5% for severe periodontitis. The estimates ranged from −4.19 to −2.93 for mild periodontitis, −1.40 to −0.42 for moderate, and −1.56 to 0.50 for severe. In all, 79.3% of the estimates for mild periodontitis, 13.1% for moderate, and <0.1% for severe had a corresponding *P* < 0.05.

As an example of the Janus effect, adjusting for the following variables results in a positive estimated association of severe periodontitis with cognitive function (β = 0.11; 95% CI, –1.32 to 1.54): age, sex, race and ethnicity, educational attainment, marital status, smoking status, physical activity, body mass index, and HbA_1c_. In contrast, adjusting for age, sex, race and ethnicity, smoking status, alcohol consumption, physical activity, health insurance status, systolic blood pressure, and total cholesterol results in a negative estimated association (β = –0.36; 95% CI, −1.78 to 1.05).

### Specification Curves

#### Cardiovascular Disease

**Figure 2.**
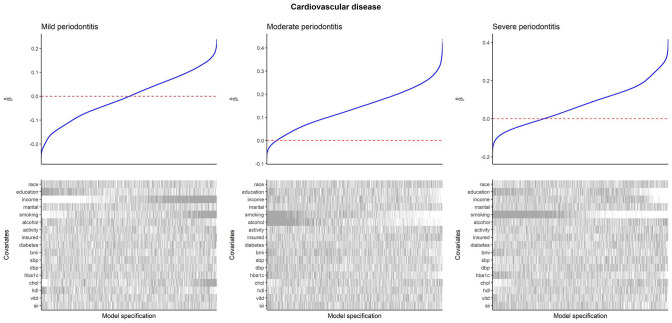
Specification curves for CVD as the outcome. Within each plot, the upper panel shows the distribution of estimated log(odds ratios) from logistic regression with CVD as the outcome and periodontitis as the exposure, and the lower panel shows the binary inclusion matrix. Gray lines in the inclusion matrix indicate that a covariate was adjusted for in the model. White lines indicate that a covariate was not adjusted for in the model. All models are adjusted for age and sex. For categorical variables, inclusion of the variable (gray line) indicates that all levels of the variable were adjusted for, and exclusion (white line) indicates that none of the levels were adjusted for. Interpretation: positive coefficients indicate that periodontitis is associated with increased odds of having CVD. CVD, cardiovascular disease.

Specification curves for CVD are presented in Figure 2 (edentulism and mean CAL results are presented in Appendix Fig. 3). For mild periodontitis, the association with CVD was typically positive when adjusting for income, smoking, and total cholesterol, but it became negative without these adjustments. For moderate periodontitis, the association was typically positive when not adjusting for smoking and alcohol consumption, but it became negative after these adjustments. For severe periodontitis, the association was typically positive when not adjusting for smoking and educational attainment, but it became negative after these adjustments.

#### Cognitive Function

**Figure 3.**
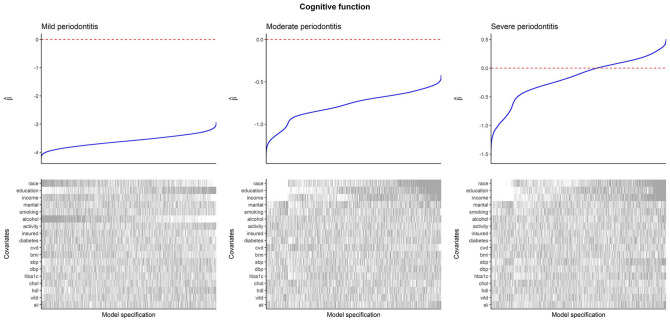
Specification curves for cognitive function as the outcome. Within each plot, the upper panel shows the distribution of estimated regression coefficients from linear regression with CERAD score as the outcome and periodontitis as the exposure, and the lower panel shows the binary inclusion matrix. Gray lines in the inclusion matrix indicate that a covariate was adjusted for in the model. White lines indicate that a covariate was not adjusted for in the model. All models are adjusted for age and sex. For categorical variables, inclusion of the variable (gray line) indicates that all levels of the variable were adjusted for, and exclusion (white line) indicates that none of the levels were adjusted for. Interpretation: negative coefficients indicate that periodontitis is associated with worse cognitive function. CERAD, Consortium to Establish a Registry for Alzheimer’s Disease.

Specification curves for cognitive function are presented in Figure 3 (edentulism and mean CAL results are presented in Appendix Fig. 4). For mild periodontitis, the association with cognitive function was always negative, strengthening with adjustment for race and ethnicity and alcohol consumption but weakening with adjustment for educational attainment. For moderate periodontitis, the association was always negative but weakened with adjustment for race and ethnicity, educational attainment, and income. For severe periodontitis, the association was typically negative when not adjusting for race and ethnicity, educational attainment, and income but became positive after these adjustments.

## Discussion

We evaluated the robustness of observational associations between periodontitis and systemic disease, using CVD and cognitive function as applied examples, by applying a multiverse analysis framework. Our findings highlight instability in these associations, suggesting that covariate selection plays an important role in determining the magnitude and direction of estimated associations. Multiverse analysis provides a transparent, reproducible method to examine how analytic decisions affect the results of a study as well as to visualize the instability underlying single-model reporting (Simonsohn et al. 2020; Vinatier et al. 2025). This framework can thus improve transparency in periodontal research. However, it addresses only model selection and misspecification, not measurement error, selection bias, or residual confounding. It also treats all model choices as equally plausible without using causal theory to prioritize specifications.

The presence of Janus effects means that observational associations may be highly prone to publication bias, as it is possible that the model specifications that produce associations in a positive or significant direction are preferentially published. This can distort the literature and mislead future investigators in that, even if they assess the entirety of the published evidence, they may arrive at the wrong conclusion regarding the association of periodontitis with systemic disease.

### Recommendations for Future Research

Some recommendations can be made for future researchers regarding best practices in selecting covariates for studies investigating the oral-systemic disease relationship (Table). An important first step would be for investigators to justify their selection of covariates. Ideally, covariates adjusted for should be based on a mechanistic understanding of the common causes of periodontitis and systemic diseases. If there are covariates that are more contentious (i.e., it could be argued that inclusion and exclusion of the variable are both valid), then some explanation of the investigators’ thought process that led to its inclusion may be helpful. We strongly suggest the use of directed acyclic graphs to make explicit the investigators’ modeling assumptions, which should then drive the use of a prespecified adjustment strategy.

**Table.** Key Considerations in Covariate Selection.

Consideration	Reason	Example
Outline hypothetical causal structure with directed acyclic graphs.	This helps to clarify the investigators’ assumptions and guides the identification of a minimal sufficient adjustment set.	A directed acyclic graph would clearly show that basic sociodemographic factors such as age, sex, and ethnicity, are common causes (i.e., confounders) of periodontitis and cardiovascular disease.
Be transparent about covariate choices.	Particularly with covariates, which could plausibly be argued as being confounders or not, some reasoning should be given why they were adjusted for.	In our present work, one could easily argue for some covariates either causing periodontitis or being caused by periodontitis (e.g., systemic immune-inflammation index). Careful consideration should be given to variables of this kind in epidemiologic analyses.
Avoid adjusting for colliders.	Colliders are variables that are common effects of the exposure and outcome. They open backdoor paths between variables when they are adjusted for, resulting in bias.	Having a diagnosis of periodontitis may cause an individual to visit the dentist more frequently. Being of lower socioeconomic status may cause an individual to visit the dentist less frequently. Therefore, conditioning on dental visits alters the association between periodontitis and socioeconomic status, which must then be accounted for.
Avoid adjusting for mediators, unless testing for underlying mechanisms of the association.	Adjusting for mediators blocks part of the association between the exposure and outcome.	It is thought that bacteria in the oral cavity can be transported via systemic circulation to the heart. Therefore, adjusting for bacterial load will block one of the pathways through which periodontitis is hypothesized to affect cardiovascular health.
Do not rely on statistically driven covariate selection.	Data-driven methods, such as stepwise regression and change in regression coefficient, do not account for the underlying biological mechanisms and are inherently influenced by the size of the dataset.	With a small sample size, there may be a null association between a covariate and the outcome, but then this association may become significant with a large sample size. Using statistically driven criteria will lead to the same variable being adjusted for in one dataset and not adjusted for in the other dataset.
Check for residual confounding.	There may be confounders that were unmeasured, unknown, or subject to measurement error.	Investigators can quantify how strong an unmeasured confounder would need to be to explain away the observed association through E-values. Alternative analyses can also be used, such as instrumental variables or negative controls.
Conduct sensitivity analyses.	The robustness of estimates to alternative modeling assumptions should be tested, where appropriate (this may overlap with checking for residual confounding).	Sensitivity analyses are question specific and can include • Adjusting for alternative sets of confounders • Subgroup analyses among different levels of the strongest confounders • Negative control exposures and/or outcomes

**Figure 4.**
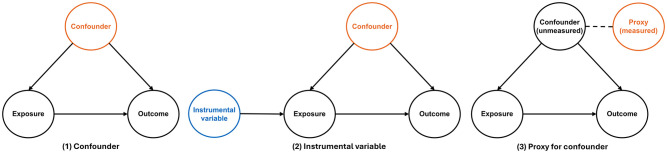
Directed acyclic graphs show the disjunctive cause criterion: (1) confounding, (2) instrumental variables, and (3) proxies for unmeasured confounding. Solid arrows indicate that one variable causally influences another, with the direction of the arrow representing the assumed causal relationship. Dashed lines indicate statistical association (i.e., correlation), without implying a direct causal effect. Orange indicates that a variable should be adjusted for, and blue indicates that a variable should not be adjusted for.

If researchers have complete knowledge of a causal diagram for common causes, they can apply the backdoor path criterion to identify a set of covariates along backdoor paths from the exposure to the outcome, which would be enough to adjust for confounding. However, comprehensive knowledge is often unavailable, but there is at least some understanding of which preexposure covariates cause the exposure and/or the outcome. Here, the disjunctive cause criterion can be used to select an appropriate set of covariates (VanderWeele and Shpitser 2011; VanderWeele 2019). This postulates that sufficient control for confounding can be achieved by 1) controlling for each covariate that is a cause of the exposure, outcome, or both; 2) excluding from this set any variable known to be an instrumental variable for the exposure; and 3) including as a covariate any proxy for an unmeasured variable that is a common cause of exposure and outcome (Lash et al. 2021). These ideas are presented in Figure 4, with additional discussion in Appendix Figure 5. In most cases, purely data-driven methods for covariate selection, such as stepwise regression or the change in regression coefficient, will not be appropriate if one wishes to draw causal inferences on the relationship between periodontitis and systemic health (Hernan and Robins 2025).

Investigators should consider study designs that are more appropriate for evaluating hypothesized causal relationships. A preference for longitudinal studies with repeated measures is important to elucidate the temporal association between periodontitis and systemic disease. Of course, such studies are often expensive and logistically challenging, but the insights that they can provide are invaluable. From an analytic perspective, several techniques have been described in the causal inference literature, such as the use of instrumental variables and negative control exposures (Lipsitch et al. 2010; Baiocchi et al. 2014). These methods complement traditional covariate adjustment by leveraging external sources of variation or exploiting assumptions that can help detect or adjust for unmeasured confounding. Replicating findings with diverse study methodologies and analytic approaches can enhance their credibility and build a more robust body of evidence (Lawlor et al. 2016).

Finally, investigators carrying out systematic reviews must be transparent regarding the set of covariates that they consider essential to adjust for confounding. The comparability domain in the widely used Newcastle-Ottawa scale suggests assigning a maximum of 2 stars to a study if it controls for the first and second most important factors (Stang 2010). In practice, this means that any periodontal-systemic disease study adjusting for sex and smoking may be considered at low risk of bias when other covariates, such as age, diabetes, and socioeconomic conditions, are theoretically also important to adjust for. Systematic reviewers should clarify whether pooled estimates correspond to crude, partially, or fully adjusted associations and whether these associations use comparable adjustment sets.

### Study Limitations

First, we focus here on bias due to confounding, but there are other sources, such as biases that arise from participant selection, measurement error, and residual confounding. Second, we focus on only 2 outcomes, but we expect that similar concerns will be present in other systemic diseases. Third, we did not include interactions between covariates, some of which might be plausible. Fourth, we consider all model choices as equally plausible, but using domain knowledge can help to cut out some of the less plausible models. Fifth, there may be important dimensions of information missing in the covariates, such as a lack of information on duration and intensity of cigarette smoking and diabetes. This leads to our final point: we have addressed here the problem of covariate selection, but there are many other choices that must be made when analyzing observational data. These include how a covariate is defined and whether it is treated as continuous or categorical, as well as the methods for addressing collinear covariates and the different definitions for exposure and outcome. This just emphasizes our key point: researchers have a huge number of decisions when analyzing observational data, and these should be made in a principled manner.

## Conclusion

This study demonstrates that estimates of association between periodontitis and systemic disease can be sensitive to model specification, particularly with respect to selection of covariates. Some recommendations are presented for future research.

## Author Contributions

N.Z. Bashir, contributed to conception, design, data acquisition, analysis, and interpretation, drafted and critically revised the manuscript; B.A.R. Woolf, S. Burgess, contributed to data interpretation, drafted and critically revised the manuscript; E. Bernabé, contributed to conception, design, data interpretation, drafted and critically revised the manuscript. All authors gave final approval and agree to be accountable for all aspects of the work.

## Supplemental Material

sj-docx-1-jdr-10.1177_00220345251356469 – Supplemental material for Periodontitis and Systemic Disease: The Impact of Covariate Selection Supplemental material, sj-docx-1-jdr-10.1177_00220345251356469 for Periodontitis and Systemic Disease: The Impact of Covariate Selection by N.Z. Bashir, B.A.R. Woolf, S. Burgess and E. Bernabé in Journal of Dental Research

## References

[bibr1-00220345251356469] Akinkugbe AA Saraiya VM Preisser JS Offenbacher S Beck JD . 2015. Bias in estimating the cross-sectional smoking, alcohol, obesity and diabetes associations with moderate-severe periodontitis in the atherosclerosis risk in communities study: comparison of full versus partial-mouth estimates. J Clin Periodontol. 42(7):609–621. 26076661 10.1111/jcpe.12425 PMC4509916

[bibr2-00220345251356469] Alhassani AA. 2023. The influence of periodontitis case definition on the association between periodontal disease and glycaemic status. Community Dent Oral Epidemiol. 51(6):1100–1108. 36601914 10.1111/cdoe.12839

[bibr3-00220345251356469] Alshihayb TS Kaye EA Zhao Y Leone CW Heaton B. 2020. The impact of periodontitis exposure misclassification bias from partial-mouth measurements on association with diabetes and cardiovascular disease. J Clin Periodontol. 47(12):1457–1465. 32990981 10.1111/jcpe.13376

[bibr4-00220345251356469] Baelum V Lopez R. 2003. Defining and classifying periodontitis: need for a paradigm shift? Eur J Oral Sci. 111(1):2–6. 12558801 10.1034/j.1600-0722.2003.00014.x

[bibr5-00220345251356469] Baiocchi M Cheng J Small DS . 2014. Instrumental variable methods for causal inference. Stat Med. 33(13):2297–2340. 24599889 10.1002/sim.6128 PMC4201653

[bibr6-00220345251356469] Beck JD Offenbacher S. 2002. Relationships among clinical measures of periodontal disease and their associations with systemic markers. Ann Periodontol. 7(1):79–89. 16013220 10.1902/annals.2002.7.1.79

[bibr7-00220345251356469] Chapple ILC Hirschfeld J Cockwell P Dietrich T Sharma P . 2024. Interplay between periodontitis and chronic kidney disease. Nat Rev Nephrol. 21(4):226–240. 39658571 10.1038/s41581-024-00910-5

[bibr8-00220345251356469] Chen CK Wu YT Chang YC . 2017. Association between chronic periodontitis and the risk of Alzheimer’s disease: a retrospective, population-based, matched-cohort study. Alzheimers Res Ther. 9(1):56. doi:10.1186/s13195-017-0282-6 28784164 PMC5547465

[bibr9-00220345251356469] Chen TC Clark J Riddles MK Mohadjer LK Fakhouri THI . 2020. National Health and Nutrition Examination Survey, 2015–2018: sample design and estimation procedures. Vital Health Stat 2. (184):1–35. 33663649

[bibr10-00220345251356469] Choi S Kim K Chang J Kim SM Kim SJ Cho HJ Park SM . 2019. Association of chronic periodontitis on Alzheimer’s disease or vascular dementia. J Am Geriatr Soc. 67(6):1234–1239. 30874308 10.1111/jgs.15828

[bibr11-00220345251356469] Demmer RT Desvarieux M. 2006. Periodontal infections and cardiovascular disease: the heart of the matter. J Am Dent Assoc. 137 Suppl:14S–20S. 10.14219/jada.archive.2006.0402 17012731

[bibr12-00220345251356469] Eke PI Page RC Wei L Thornton-Evans G Genco RJ . 2012. Update of the case definitions for population-based surveillance of periodontitis. J Periodontol. 83(12):1449–1454. 22420873 10.1902/jop.2012.110664 PMC6005373

[bibr13-00220345251356469] Fillenbaum GG van Belle G Morris JC Mohs RC Mirra SS Davis PC Tariot PN Silverman JM Clark CM Welsh-Bohmer KA , et al. 2008. Consortium to Establish a Registry for Alzheimer’s Disease (CERAD): the first twenty years. Alzheimers Dement. 4(2):96–109. 18631955 10.1016/j.jalz.2007.08.005 PMC2808763

[bibr14-00220345251356469] Hernan MA Robins JM . 2025. Causal inference: what if. Boca Raton (FL): CRC Press.

[bibr15-00220345251356469] Hu B Yang X-R Xu Y Sun Y-F Sun C Guo W Zhang X Wang W-M Qiu S-J Zhou J , et al. 2014. Systemic immune-inflammation index predicts prognosis of patients after curative resection for hepatocellular carcinoma. Clin Cancer Res. 20(23):6212–6222. 25271081 10.1158/1078-0432.CCR-14-0442

[bibr16-00220345251356469] Lash TL Rothman KJ VanderWeele TJ Haneuse S. 2021. Modern epidemiology. 4th ed. Riverwoods (IL): Wolters Kluwer. 10.1007/s10654-021-00778-w PMC8416883 34216355

[bibr17-00220345251356469] Lawlor DA Tilling K Davey Smith G. 2016. Triangulation in aetiological epidemiology. Int J Epidemiol. 45(6):1866–1886. 28108528 10.1093/ije/dyw314 PMC5841843

[bibr18-00220345251356469] Lipsitch M Tchetgen Tchetgen E Cohen T. 2010. Negative controls: a tool for detecting confounding and bias in observational studies. Epidemiology. 21(3):383–388. 20335814 10.1097/EDE.0b013e3181d61eeb PMC3053408

[bibr19-00220345251356469] Lonsdorf TB Klingelhöfer-Jens M Andreatta M Beckers T Chalkia A Gerlicher A Jentsch VL Meir Drexler S Mertens G Richter J , et al. 2019. Navigating the garden of forking paths for data exclusions in fear conditioning research. Elife. 8:e52465. doi:10.7554/elife.52465 PMC6989118 31841112

[bibr20-00220345251356469] Ma J Li K. 2023. Systemic immune-inflammation index is associated with coronary heart disease: a cross-sectional study of NHANES 2009–2018. Front Cardiovasc Med. 10:1199433. doi:10.3389/fcvm.2023.1199433 37485261 PMC10361751

[bibr21-00220345251356469] Manau C Echeverria A Agueda A Guerrero A Echeverria JJ . 2008. Periodontal disease definition may determine the association between periodontitis and pregnancy outcomes. J Clin Periodontol. 35(5):385–397. 18341599 10.1111/j.1600-051X.2008.01222.x

[bibr22-00220345251356469] Nascimento GG Leite FRM Mesquita CM Vidigal MTC Borges GH Paranhos LR . 2023. Confounding in observational studies evaluating the association between Alzheimer’s disease and periodontal disease: a systematic review. Heliyon. 9(4):e15402. doi:10.1016/j.heliyon.2023.e15402 PMC10147971 37128313

[bibr23-00220345251356469] Preshaw PM Alba AL Herrera D Jepsen S Konstantinidis A Makrilakis K Taylor R. 2012. Periodontitis and diabetes: a two-way relationship. Diabetologia. 55(1):21–31. 22057194 10.1007/s00125-011-2342-y PMC3228943

[bibr24-00220345251356469] Sanz M Marco Del Castillo A Jepsen S Gonzalez-Juanatey JR D’Aiuto F Bouchard P Chapple I Dietrich T Gotsman I Graziani F , et al. 2020. Periodontitis and cardiovascular diseases: consensus report. J Clin Periodontol. 47(3):268–288. 32011025 10.1111/jcpe.13189 PMC7027895

[bibr25-00220345251356469] Simonsohn U Simmons JP Nelson LD . 2020. Specification curve analysis. Nat Hum Behav. 4(11):1208–1214. 32719546 10.1038/s41562-020-0912-z

[bibr26-00220345251356469] Stang A. 2010. Critical evaluation of the Newcastle-Ottawa scale for the assessment of the quality of nonrandomized studies in meta-analyses. Eur J Epidemiol. 25(9):603–605. 20652370 10.1007/s10654-010-9491-z

[bibr27-00220345251356469] Steegen S Tuerlinckx F Gelman A Vanpaemel W. 2016. Increasing transparency through a multiverse analysis. Perspect Psychol Sci. 11(5):702–712. 27694465 10.1177/1745691616658637

[bibr28-00220345251356469] VanderWeele TJ. 2019. Principles of confounder selection. Eur J Epidemiol. 34(3):211–219. 30840181 10.1007/s10654-019-00494-6 PMC6447501

[bibr29-00220345251356469] VanderWeele TJ Shpitser I. 2011. A new criterion for confounder selection. Biometrics. 67(4):1406–1413. 21627630 10.1111/j.1541-0420.2011.01619.x PMC3166439

[bibr30-00220345251356469] Vinatier C Hoffmann S Patel C DeVito NJ Cristea IA Tierney B Ioannidis JPA Naudet F. 2025. What is the vibration of effects? BMJ Evid Based Med. 30(1):61–65. 10.1136/bmjebm-2023-112747 PMC11874424 38997151

