# Supplementary material for: Periodontitis and Systemic Disease: The Impact of Covariate Selection
Source: J Dent Res. 2025 Jul 17;105(1):59–66. doi: 10.1177/00220345251356469 (PMC12701905; doi:10.1177/00220345251356469)
Supplement: sj-docx-1-jdr-10.1177_00220345251356469 – Supplemental material for Periodontitis and Systemic Disease: The Impact of Covariate Selection [file sj-docx-1-jdr-10.1177_00220345251356469.docx]

**APPENDIX**

**Periodontitis and Systemic Disease: The Impact of Covariate Selection**

**Authors**

Nasir Z. Bashir ^1^, Benjamin A.R. Woolf ^1,2,3^, Stephen Burgess ^1,4^, Eduardo Bernabé ^5^

^1^ MRC Biostatistics Unit, University of Cambridge, Cambridge, United Kingdom

^2^ MRC Integrative Epidemiology Unit, University of Bristol, Bristol, United Kingdom

^3^ School of Psychological Science, University of Bristol, Bristol, United Kingdom

^4^ Cardiovascular Epidemiology Unit, University of Cambridge, Cambridge, United Kingdom

^5^ Institute of Dentistry, Queen Mary University of London, London, United Kingdom

**Corresponding Author**

Mr. Nasir Bashir

MRC Biostatistics Unit

University of Cambridge

Cambridge

United Kingdom

CB2 0SR

nzb22@cam.ac.uk

**Appendix Table 1.** Characteristics of the individuals included in the CVD analyses.

| Characteristic | Periodontal status | | | | |
| --- | --- | --- | --- | --- | --- |
|  | None  (*n* = 3595) | Mild  (*n* = 135) | Moderate  (*n* = 2470) | Severe  (*n* = 752) | Edentate  (*n* = 685) |
| Age, years | 46.0  [37.0, 59.0] | 41.0  [34.0, 50.5] | 56.0  [44.0, 67.0] | 56.0  [48.0, 64.0] | 70.0  [61.0, 77.0] |
| Sex |  |  |  |  |  |
| Men | 1472 (40.9) | 74 (54.8) | 1325 (53.6) | 532 (70.7) | 340 (49.6) |
| Women | 2123 (59.1) | 61 (45.2) | 1145 (46.4) | 220 (29.3) | 345 (50.4) |
| Race / ethnicity |  |  |  |  |  |
| Non-Hispanic White | 1709 (47.5) | 46 (34.1) | 852 (34.5) | 193 (25.7) | 341 (49.8) |
| Non-Hispanic Black | 627 (17.4) | 32 (23.7) | 625 (25.3) | 266 (35.4) | 185 (27.0) |
| Mexican American | 661 (18.4) | 34 (25.2) | 626 (25.3) | 185 (24.6) | 89 (13.0) |
| Other | 598 (16.6) | 23 (17.0) | 367 (14.9) | 108 (14.4) | 70 (10.2) |
| *Missing, n (%)* | *0 (0)* | *0 (0)* | *0 (0)* | *0 (0)* | *0 (0)* |
| Educational attainment |  |  |  |  |  |
| Below high school | 498 (13.9) | 17 (12.6) | 700 (28.4) | 266 (35.4) | 312 (45.6) |
| High school | 1715 (47.7) | 85 (63.0) | 1295 (52.5) | 394 (52.4) | 328 (48.0) |
| More than high school | 1381 (38.4) | 33 (24.4) | 472 (19.1) | 92 (12.2) | 44 (6.4) |
| *Missing, n (%)* | *1 (<0.1)* | *0 (0)* | *3 (0.1)* | *0 (0)* | *1 (0.1)* |
| Income-to-poverty ratio | 3.2  [1.4, 5.0] | 2.3  [1.3, 4.1] | 1.8  [1.0, 3.5] | 1.6  [0.9, 3.0] | 1.2  [0.9, 2.3] |
| *Missing, n (%)* | *237 (6.6)* | *15 (11.1)* | *211 (8.5)* | *82 (10.9)* | *60 (8.8)* |
| Marital status |  |  |  |  |  |
| Living as married | 2454 (68.3) | 92 (68.1) | 1497 (60.7) | 437 (58.1) | 304 (44.4) |
| Separated | 707 (19.7) | 20 (14.8) | 697 (28.2) | 212 (28.2) | 316 (46.1) |
| Never married | 433 (12.0) | 23 (17.0) | 274 (11.1) | 103 (13.7) | 65 (9.5) |
| *Missing, n (%)* | *1 (<0.1)* | *0 (0)* | *2 (0.1)* | *0 (0)* | *0 (0)* |
| Smoking status |  |  |  |  |  |
| Never smoked | 2329 (64.8) | 100 (74.1) | 1223 (49.6) | 291 (38.7) | 224 (32.7) |
| Former smoker | 819 (22.8) | 19 (14.1) | 683 (27.7) | 197 (26.2) | 250 (36.5) |
| Current smoker | 447 (12.4) | 16 (11.9) | 560 (22.7) | 263 (35.0) | 211 (30.8) |
| *Missing, n (%)* | *0 (0)* | *0 (0)* | *4 (0.2)* | *1 (0.1)* | *0 (0)* |
| Alcohol consumption, drinks/day | 0.1  [0.0, 0.4] | 0.1  [0.0, 0.4] | 0.0  [0.0, 0.5] | 0.1  [0.0, 1.0] | 0.0  [0.0, 0.1] |
| *Missing, n (%)* | *541 (15.0)* | *24 (17.8)* | *543 (22.0)* | *165 (21.9)* | *232 (33.9)* |
| Physical activity, MET-hrs/week | 17.3  [2.7, 52.0] | 16.0  [3.0, 52.0] | 16.0  [0.0, 56.0] | 18.5  [0.0, 76.5] | 2.0  [0.0, 22.0] |
| *Missing, n (%)* | *8 (0.2)* | *0 (0)* | *9 (0.4)* | *4 (0.5)* | *2 (0.3)* |
| Health insurance status |  |  |  |  |  |
| Uninsured | 600 (16.7) | 36 (26.7) | 611 (24.8) | 244 (32.4) | 78 (11.4) |
| Insured | 2995 (83.3) | 99 (73.3) | 1857 (75.2) | 508 (67.6) | 606 (88.6) |
| *Missing, n (%)* | *0 (0)* | *0 (0)* | *2 (0.1)* | *0 (0)* | *1 (0.1)* |
| Self-reported diabetes |  |  |  |  |  |
| No | 3274 (91.1) | 120 (88.9) | 2027 (82.1) | 629 (83.8) | 495 (72.4) |
| Yes | 319 (8.9) | 15 (11.1) | 443 (17.9) | 122 (16.2) | 189 (27.6) |
| *Missing, n (%)* | *2 (0.1)* | *0 (0)* | *0 (0)* | *1 (0.1)* | *1 (0.1)* |
| BMI, kg/m^2^ | 27.70  [24.3, 32.1] | 28.7  [25.7, 33.3] | 28.3  [24.7, 32.9] | 27.7  [24.4, 31.9] | 27.70  [24.5, 31.6] |
| *Missing, n (%)* | *18 (0.5)* | *0 (0)* | *25 (1.0)* | *6 (0.8)* | *14 (2.0)* |
| SBP, mmHg | 118.7 [110.0, 130.0] | 119.3 [110.5, 128.0] | 125.0 [114.0, 137.3] | 126.7 [115.3, 141.3] | 129.3 [117.3, 143.5] |
| *Missing, n (%)* | *123 (3.4)* | *5 (3.7)* | *104 (4.2)* | *31 (4.1)* | *29 (4.2)* |
| DBP, mmHg | 72.0  [65.3, 78.7] | 70.7  [63.3, 77.2] | 71.3  [64.0, 78.7] | 73.3  [66.0, 80.7] | 68.0  [58.7, 74.7] |
| *Missing, n (%)* | *123 (3.4)* | *5 (3.7)* | *104 (4.2)* | *31 (4.1)* | *29 (4.2)* |
| HbA1c, % | 5.5  [5.2, 5.8] | 5.5  [5.2, 5.8] | 5.7  [5.4, 6.1] | 5.70  [5.0, 6.1] | 5.8  [5.5, 6.3] |
| *Missing, n (%)* | *119 (3.3)* | *2 (1.5)* | *94 (3.8)* | *34 (4.5)* | *31 (4.5)* |
| Total cholesterol, mmol/L | 5.0  [4.3, 5.7] | 5.0  [4.4, 5.6] | 4.97  [4.3, 5.7] | 5.0  [4.4, 5.7] | 4.7  [4.0, 5.6] |
| *Missing, n (%)* | *155 (4.3)* | *4 (3.0)* | *123 (5.0)* | *44 (5.9)* | *54 (7.9)* |
| HDL cholesterol, mmol/L | 1.3  [1.1, 1.6] | 1.3  [1.0, 1.5] | 1.3  [1.1, 1.6] | 1.2  [1.1, 1.5] | 1.2  [1.0, 1.5] |
| *Missing, n (%)* | *155 (4.3)* | *4 (3.0)* | *123 (5.0)* | *44 (5.9)* | *54 (7.9)* |
| Vitamin D, nmol/L | 66.5  [50.1, 84.5] | 56.1  [41.2, 76.3] | 62.0  [43.9, 80.1] | 56.7  [39.7, 72.7] | 67.6  [47.6, 89.9] |
| *Missing, n (%)* | *139 (3.9)* | *3 (2.2)* | *110 (4.5)* | *37 (4.9)* | *44 (6.4)* |
| SII | 441.9 [318.8, 613.9] | 462.2 [352.8, 644.0] | 444.7 [318.9, 634.0] | 449.8 [312.2, 659.6] | 472.1 [313.8, 699.7] |
| *Missing, n (%)* | *117 (3.3)* | *2 (1.5)* | *89 (3.6)* | *34 (4.5)* | *33 (4.8)* |
| CVD |  |  |  |  |  |
| No | 3421 (95.2) | 129 (95.6) | 2234 (90.4) | 689 (91.6) | 507 (74.0) |
| Yes | 174 (4.8) | 6 (4.4) | 236 (9.6) | 63 (8.4) | 178 (26.0) |

BMI: body mass index; CVD: cardiovascular disease; HbA1c: glycated hemoglobin; HDL; high-density lipoprotein; MET-hrs: metabolic equivalent hours; SII: systemic immune-inflammation index.

Categorical variables are reported as the number of participants (%). Continuous variables are reported as median [interquartile range]. Summary statistics for continuous and categorical variables are calculated amongst individuals without missing data, for that variable.

Any discrepancy between the sum of subgroups and total sample is due to rounding error. All values are computed based on individuals with complete data for age, sex, and CVD.

**Appendix Table 2.** Characteristics of the individuals included in the cognitive function analyses.

| Characteristic | Periodontal status | | | | |
| --- | --- | --- | --- | --- | --- |
|  | None  (*n* = 823) | Mild  (*n* = 14) | Moderate  (*n* = 986) | Severe  (*n* = 276) | Edentate  (*n* = 469) |
| Age, years | 67.0  [63.0, 74.0] | 68.0  [65.0, 71.0] | 68.5  [64.0, 76.0] | 65.0  [62.0, 70.0] | 72.0  [66.0, 79.0] |
| Sex |  |  |  |  |  |
| Men | 310 (37.7) | 7 (50.0) | 528 (53.5) | 202 (73.2) | 234 (49.9) |
| Women | 513 (62.3) | 7 (50.0) | 458 (46.5) | 74 (26.8) | 235 (50.1) |
| Race / ethnicity |  |  |  |  |  |
| Non-Hispanic White | 467 (56.7) | 11 (78.6) | 409 (41.5) | 63 (22.8) | 218 (46.5) |
| Non-Hispanic Black | 137 (16.6) | 0 (0.0) | 251 (25.5) | 104 (37.7) | 151 (32.2) |
| Mexican American | 136 (16.5) | 2 (14.3) | 218 (22.1) | 84 (30.4) | 62 (13.2) |
| Other | 83 (10.1) | 1 (7.1) | 108 (11.0) | 25 (9.1) | 38 (8.1) |
| *Missing, n (%)* | *0 (0)* | *0 (0)* | *0 (0)* | *0 (0)* | *0 (0)* |
| Educational attainment |  |  |  |  |  |
| Below high school | 134 (16.3) | 1 (7.1) | 261 (26.5) | 95 (34.4) | 211 (45.1) |
| High school | 432 (52.5) | 11 (78.6) | 492 (50.0) | 146 (52.9) | 225 (48.1) |
| More than high school | 257 (31.2) | 2 (14.3) | 231 (23.5) | 35 (12.7) | 32 (6.8) |
| *Missing, n (%)* | *0 (0)* | *0 (0)* | *2 (0.2)* | *0 (0)* | *1 (0.2)* |
| Income-to-poverty ratio | 3.1  [1.6, 5.0] | 3.1  [1.7, 5.0] | 2.1  [1.2, 3.9] | 1.7  [1.0, 3.0] | 1.3  [0.9, 2.6] |
| *Missing, n (%)* | *64 (7.8)* | *3 (21.4)* | *64 (6.5)* | *32 (11.6)* | *48 (10.2)* |
| Marital status |  |  |  |  |  |
| Living as married | 522 (63.5) | 11 (78.6) | 559 (56.9) | 160 (58.0) | 204 (43.5) |
| Separated | 245 (29.8) | 2 (14.3) | 373 (37.9) | 93 (33.7) | 237 (50.5) |
| Never married | 55 (6.7) | 1 (7.1) | 51 (5.2) | 23 (8.3) | 28 (6.0) |
| *Missing, n (%)* | *1 (0.1)* | *0 (0)* | *0 (0)* | *0 (0)* | *0 (0)* |
| Smoking status |  |  |  |  |  |
| Never smoked | 506 (61.5) | 10 (71.4) | 473 (48.1) | 111 (40.2) | 158 (33.7) |
| Former smoker | 271 (32.9) | 3 (21.4) | 387 (39.4) | 94 (34.1) | 206 (43.9) |
| Current smoker | 46 (5.6) | 1 (7.1) | 123 (12.5) | 71 (25.7) | 105 (22.4) |
| *Missing, n (%)* | *0 (0)* | *0 (0)* | *3 (0.3)* | *0 (0)* | *0 (0)* |
| Alcohol consumption, drinks/day | 0.0  [0.0, 0.3] | 0.0  [0.0, 0.1] | 0.0  [0.00, 0.3] | 0.  [0.0, 1.1] | 0.0  [0.0, 0.1] |
| *Missing, n (%)* | *110 (13.4)* | *0 (0)* | *198 (20.1)* | *58 (21)* | *150 (32)* |
| Physical activity, MET-hrs/week | 10.0  [0.0, 36.0] | 5.0  [1.3, 17.7] | 12.0  [0.0, 33.3] | 10.0  [0.0, 40.0] | 2.0  [0.0, 21.5] |
| *Missing, n (%)* | *2 (0.2)* | *2 (0.2)* | *2 (0.2)* | *2 (0.2)* | *2 (0.2)* |
| Health insurance status |  |  |  |  |  |
| Uninsured | 54 (6.6) | 0 (0.0) | 86 (8.7) | 45 (16.3) | 31 (6.6) |
| Insured | 769 (93.4) | 14 (100.0) | 900 (91.3) | 231 (83.7) | 436 (93.4) |
| *Missing, n (%)* | *0 (0)* | *0 (0)* | *0 (0)* | *0 (0)* | *2 (0.4)* |
| Diabetes |  |  |  |  |  |
| No | 679 (82.5) | 12 (85.7) | 726 (73.7) | 220 (79.7) | 327 (69.9) |
| Yes | 144 (17.5) | 2 (14.3) | 259 (26.3) | 56 (20.3) | 141 (30.1) |
| *Missing, n (%)* | *0 (0)* | *0 (0)* | *1 (0.1)* | *0 (0)* | *1 (0.2)* |
| CVD |  |  |  |  |  |
| No | 700 (85.3) | 12 (85.7) | 824 (84.0) | 240 (87.3) | 337 (72.2) |
| Yes | 121 (14.7) | 2 (14.3) | 157 (16.0) | 35 (12.7) | 130 (27.8) |
| *Missing, n (%)* | *2 (0.2)* | *0 (0)* | *5 (0.5)* | *1 (0.4)* | *2 (0.4)* |
| BMI, kg/m^2^ | 27.9  [24.8, 32.2] | 29.5  [26.8, 35.1] | 28.0  [24.6, 32.1] | 27.5  [24.3, 31.4] | 27.9  [24.8, 31.7] |
| *Missing, n (%)* | *2 (0.2)* | *0 (0)* | *10 (1)* | *4 (1.4)* | *13 (2.8)* |
| SBP, mmHg | 129.3  [118.7, 142.7] | 129.3 [124.8, 141.0] | 131.3 [119.7, 143.3] | 132.7 [120.7, 147.3] | 132.0 [120.0, 146.7] |
| *Missing, n (%)* | *22 (2.7)* | *0 (0)* | *27 (2.7)* | *5 (1.8)* | *16 (3.4)* |
| DBP, mmHg | 70.0  [62.7, 76.7] | 67.3  [62.0, 75.0] | 68.7  [60.7, 76.0] | 70.7  [62.7, 78.0] | 66.0  [57.3, 74.0] |
| *Missing, n (%)* | *22 (2.7)* | *0 (0)* | *27 (2.7)* | *5 (1.8)* | *16 (3.4)* |
| HbA1c, % | 5.7  [5.4, 6.1] | 5.8  [5.5, 6.3] | 5.8  [5.5, 6.3] | 5.8  [5.5, 6.2] | 5.9  [5.5, 6.4] |
| *Missing, n (%)* | *25 (3)* | *0 (0)* | *34 (3.4)* | *12 (4.3)* | *16 (3.4)* |
| Total cholesterol, mmol/L | 5.0  [4.3, 5.7] | 4.8  [4.4, 5.4] | 4.9  [4.2, 5.6] | 4.9  [4.2, 5.7] | 4.6  [4.0, 5.5] |
| *Missing, n (%)* | *41 (5)* | *0 (0)* | *41 (4.2)* | *14 (5.1)* | *33 (7)* |
| HDL cholesterol, mmol/L | 1.4  [1.1, 1.7] | 1.2  [1.0, 1.4] | 1.3  [1.1, 1.6] | 1.3  [1.1, 1.6] | 1.3  [1.1, 1.5] |
| *Missing, n (%)* | *41 (5)* | *0 (0)* | *41 (4.2)* | *14 (5.1)* | *33 (7)* |
| Vitamin D, nmol/L | 79.8  [61.3, 98.2] | 69.8  [41.5, 93.0] | 72.2  [53.3, 91.3] | 60.6  [41.7, 82.5] | 70.8  [50.3, 92.8] |
| *Missing, n (%)* | *34 (4.1)* | *0 (0)* | *38 (3.9)* | *13 (4.7)* | *24 (5.1)* |
| SII | 445.9  [327.3, 608.8] | 622.2  [366.1, 700.1] | 445.6  [313.6, 653.1] | 424.8  [295.1, 658.2] | 473.3  [318.6, 701.1] |
| *Missing, n (%)* | *22 (2.7)* | *0 (0)* | *29 (2.9)* | *12 (4.3)* | *19 (4.1)* |
| CERAD score | 27.0  [22.0, 31.0] | 25.0  [22.5, 27.0] | 25.0  [20.0, 29.0] | 24.0  [20.0, 29.0] | 23.0  [18.0, 27.0] |

BMI: body mass index; CERAD: Consortium to Establish a Registry for Alzheimer’s Disease test; CVD: cardiovascular disease; HbA1c: glycated hemoglobin; HDL; high-density lipoprotein; DBP: diastolic blood pressure; MET-hrs: metabolic equivalent hours; SBP: systolic blood pressure; SII: systemic immune-inflammation index.

Categorical variables are reported as the number of participants (%). Continuous variables are reported as median [interquartile range]. Summary statistics for continuous and categorical variables are calculated amongst individuals without missing data, for that variable.

Any discrepancy between the sum of subgroups and total sample is due to rounding error. All values are computed based on individuals with complete data for age, sex, and CERAD score.


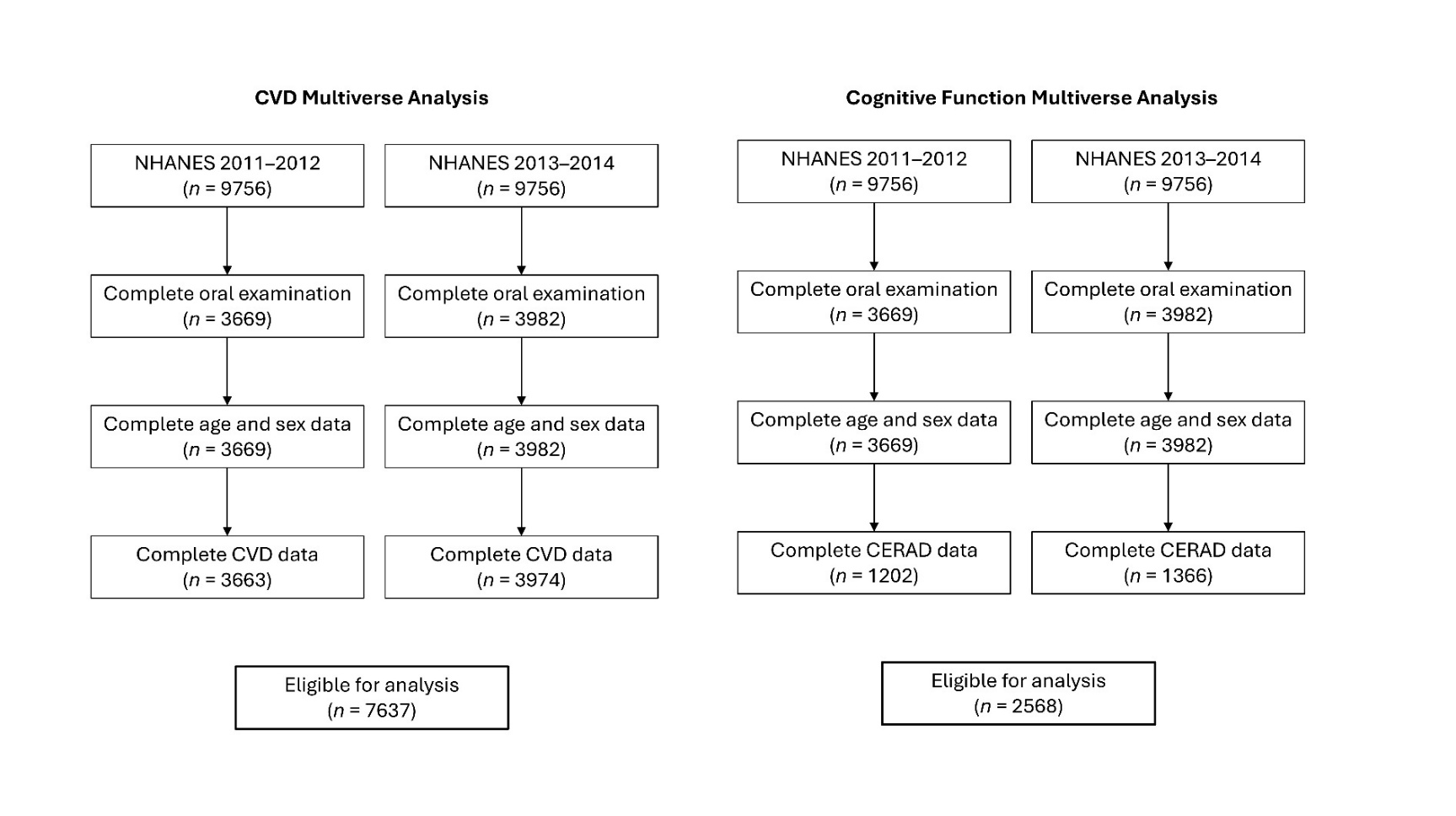
**Appendix Figure 1.** Flowchart showing procedure for selecting included participants, by NHANES study cycle.


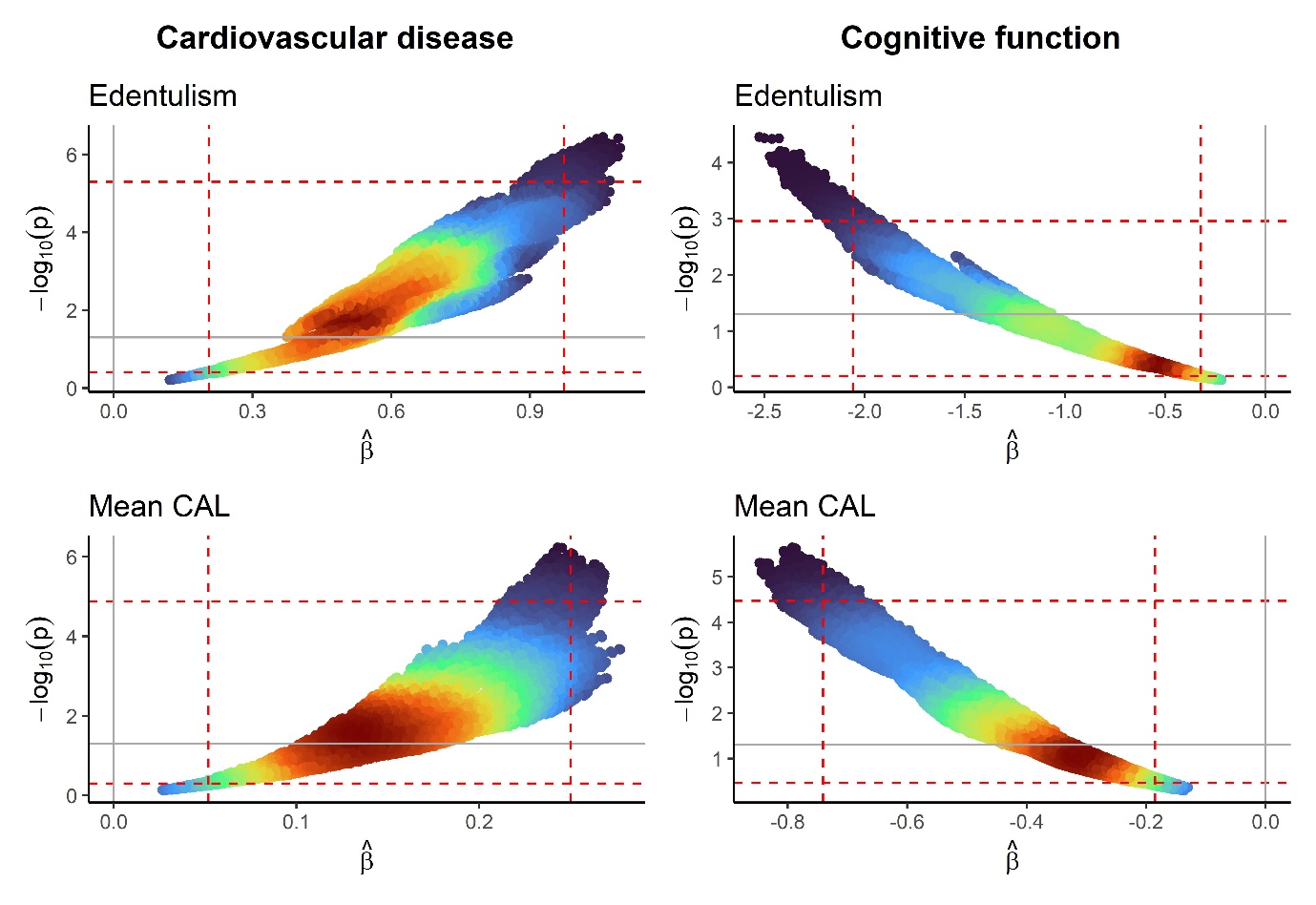
**Appendix Figure 2.** Volcano plots where each point represents the estimated regression coefficient and -log_10_(*p*-value) from one model, coloured by its density (red = high, blue = low). Vertical and horizontal dashed red lines represent the 1^st^ and 99^th^ percentiles of the estimated regression coefficient and -log_10_(*p*-value), respectively. Vertical and horizontal solid grey lines represent the null effect and nominal threshold of significance (*p* = 0.05), respectively. For edentulism, no periodontitis is the reference category. For mean CAL, the exposure corresponds to a 1mm increase in mean CAL. Left column: x-axis for CVD volcano plot is measuring the log(OR). Right column: x-axis for cognitive function volcano plot is measuring the linear regression coefficient. All models are adjusted for age and sex.

**
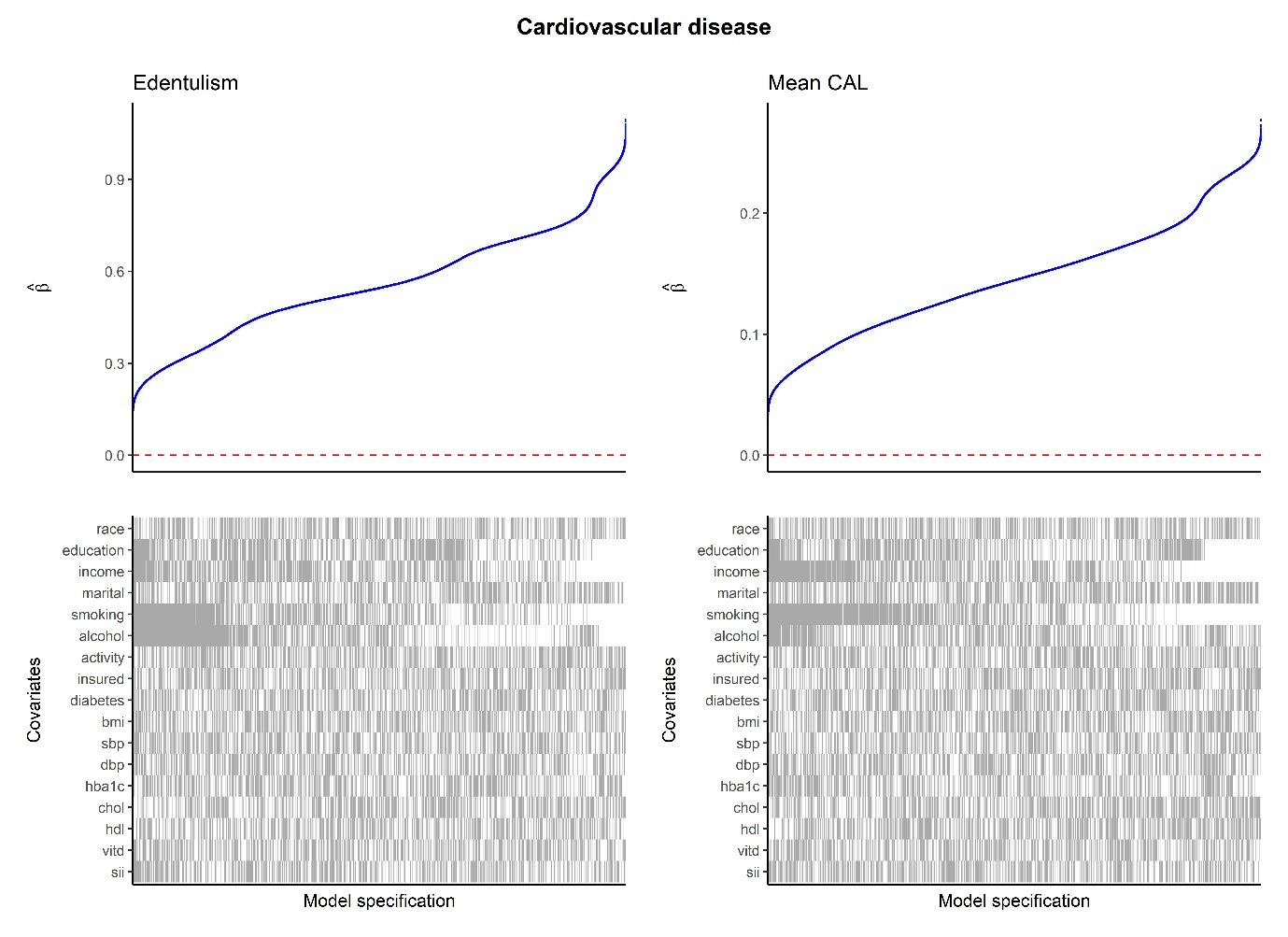
Appendix Figure 3.** Specification curves for edentulism and mean CAL, with CVD as the outcome. Within each plot, the upper panel shows the distribution of estimated log(ORs) from logistic regression with CVD as the outcome and periodontitis as the exposure, and the lower panel shows a binary inclusion matrix. Grey lines in the inclusion matrix indicate that a covariate was adjusted for in the model. White lines indicate that a covariate was not adjusted for in the model. All models are adjusted for age and sex. For categorical variables, inclusion of the variable (grey line) indicates that all levels of the variable were adjusted for, and exclusion (white line) indicates that none of the levels of the variable were adjusted for. Interpretation: positive coefficients indicate periodontitis is associated with increased odds of having CVD.

**
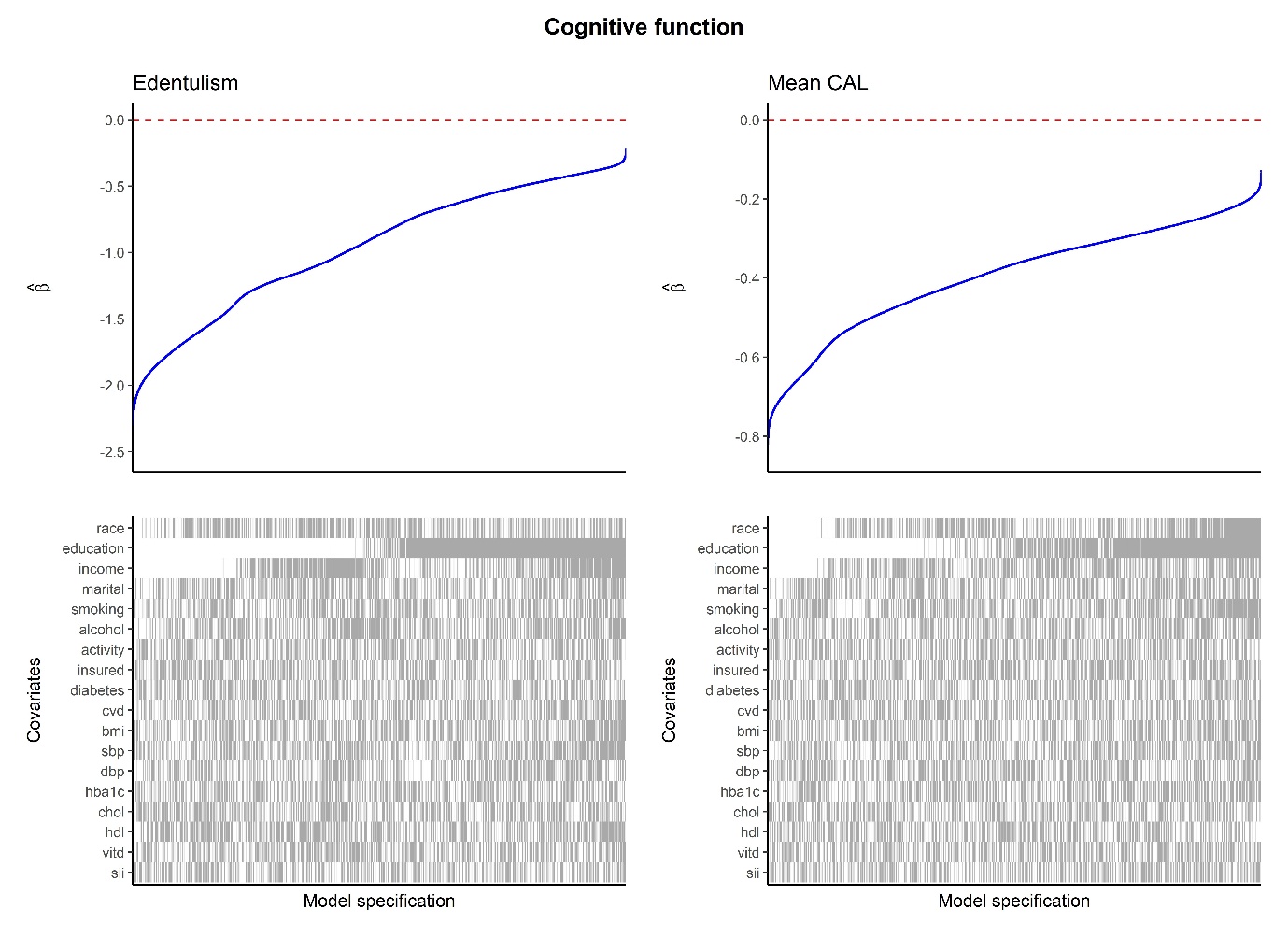
Appendix Figure 4.** Specification curves for edentulism and mean CAL, with cognitive function as the outcome. Within each plot, the upper panel shows the distribution of estimated linear regression coefficients from linear regression with CERAD score as the outcome and periodontitis as the exposure, and the lower panel shows a binary inclusion matrix. Grey lines in the inclusion matrix indicate that a covariate was adjusted for in the model. White lines indicate that a covariate was not adjusted for in the model. All models are adjusted for age and sex. For categorical variables, inclusion of the variable (grey line) indicates that all levels of the variable were adjusted for, and exclusion (white line) indicates that none of the levels of the variable were adjusted for. Interpretation: negative coefficients indicate periodontitis is associated with worse cognitive function.

**
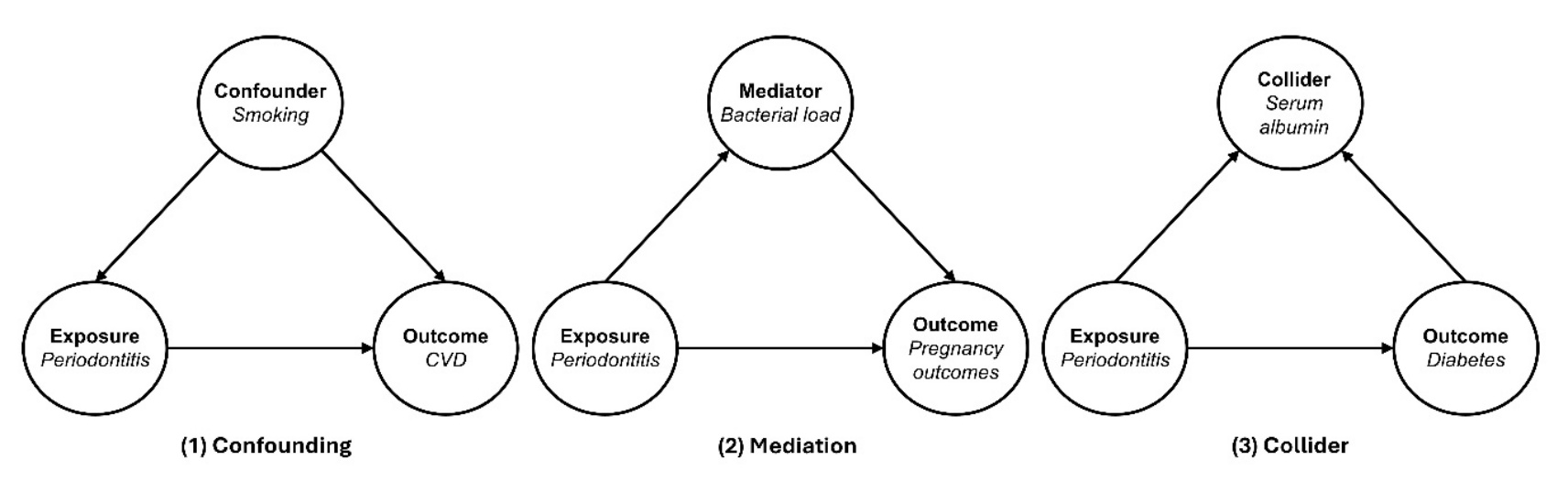
**

**Appendix Figure 5.** Directed acyclic graphs showing hypothetical causal relationships in periodontal-systemic disease relationships. Solid arrows indicate that one variable causally influences another, with the direction of the arrow representing the assumed causal relationship. In scenario 1, controlling for the confounder would be necessary to reduce bias. In scenario 2, controlling for the mediator would mean that the association between the exposure and outcome is biased, as this adjustment attenuates the component that of the association that acts through the mediator. In scenario 3, controlling for the collider would induce bias in the association between the exposure and outcome.
